# Study on the effects of exercise intervention combined with virtual reality technology on emotions and brain networks in secondary school students with depressive symptoms

**DOI:** 10.3389/fpsyt.2025.1728197

**Published:** 2026-01-30

**Authors:** Ting Peng, Jing Song, Yizhong Ren, Jinghui Yang, Yi Zhang, Aiping Chi

**Affiliations:** School of Physical Education, Shaanxi Normal University, Xi’an, Shaanxi, China

**Keywords:** brain networks, depressive symptoms, exercise intervention, mood, secondary school students, virtual reality

## Abstract

**Background:**

This study aimed to investigate the functional connectivity characteristics of brain networks in secondary school students with depressive symptoms and to analyze the effects of exercise combined with virtual reality intervention on improving brain networks and emotional states, providing a neurobiological basis for early identification and precise intervention.

**Methods:**

This study recruited 98 middle school students aged 13 to 18 as research subjects, including 50 in the subclinical depression (ScD) group and 48 in the healthy control (HC) group. The experimental intervention employed a 2×3 two-way mixed design analysis of variance (Two-way ANOVA). All exercise intervention groups underwent 15 minutes of moderate-intensity (50%-80% HRmax) power cycling training. The exercise intervention combined with virtual reality technology group completed their training in an immersive natural landscape environment. Resting-state EEG signals were recorded before and after the intervention, and emotional state changes were assessed using the Positive and Negative Affect Schedule (PANAS). The cerebral cortex was segmented into 78 regions based on the Schaefer template. Phase-locked value ( $PLV=\frac{1}{T}|\sum_{t=1}^{T}e^{i(\phi_{i}(t)-\phi_{j}(t))}|$) was used as a functional connectivity metric to quantify brain network synchrony in the theta, alpha, and beta frequency bands. Statistical comparisons were performed using independent samples t-tests and two-way analysis of variance (ANOVA).

**Results:**

Exercise intervention combined with virtual reality technology significantly improved θ and α band SMN-DMN, DAN-SN connectivity, and DMN/DAN activity (*p* < 0.05), outperforming conventional exercise. β band SMN-DMN and CEN-DMN activity increased (*p<* 0.05). The exercise intervention combined with virtual reality technology significantly increased positive emotions (t = -22.351, *p* < 0.05) and reduced negative emotions (t = 27.257, *p* < 0.001).

**Conclusion:**

Depressive symptoms in adolescents are associated with multifrequency brain network dysregulation. Combining exercise intervention with virtual reality technology (VR-EI) optimizes key brain network connectivity and activity in the theta and alpha bands through multisensory stimulation. Its mood-enhancing effects surpass those of conventional exercise, offering a promising new strategy for personalized intervention in adolescent depression.

## 1 Introduction

In recent years, depressive symptoms have shown a trend toward younger onset, with their prevalence among secondary school students continuing to rise, attracting widespread attention from both society and academia (1). A nationwide survey covering five provinces in China revealed that the prevalence rate of depressive symptoms among middle school students reached 24.5%, with a rising trend observed across higher grades (2). As adolescents in a critical developmental stage, secondary school students exhibit high neuroplasticity due to the immature integration of their prefrontal cortex, limbic system, and related functional networks. This heightened sensitivity to psychological stress and emotional fluctuations increases their susceptibility to depressive symptoms (3). Depressive symptoms represent an intermediate state that does not yet meet clinical diagnostic criteria (4). Without timely intervention, they may progressively worsen and evolve into clinical depression, imposing a profound burden on an individual’s psychological well-being and physiological functioning (5). Emotional regulation is a crucial process for secondary school students to adapt to society, maintain interpersonal relationships, and protect mental health (6). However, the immaturity of emotional regulation and cognitive control abilities during adolescence may lead young people to adopt maladaptive coping strategies such as excessive rumination, avoidance, or emotional outbursts, increasing the risk of internalizing problems like depression and anxiety, as well as externalizing problems like aggressive behavior (7). Therefore, in-depth investigation into the neural mechanisms underlying depressive symptoms in secondary school students and exploration of effective emotion regulation strategies are crucial for achieving early identification, prevention, and precise intervention.

Research on functional connectivity within brain networks offers a novel perspective for elucidating the neural mechanisms of depression. Normal brain function relies on complex, coordinated network connections and synergistic activity among different brain regions. Previous studies have demonstrated significant abnormalities in functional connectivity across multiple key brain networks in individuals with depression, including the Default Mode Network (DMN), Dorsal Attention Network (DAN), and Salience Network (SN) (8). These networks play indispensable roles in core cognitive processes such as emotion regulation, attentional control, and self-processing (9). In recent years, studies based on electroencephalography (EEG) have further revealed that neuro-oscillatory activity in individuals with depression exhibits abnormalities across multiple frequency bands. For instance, theta band (4–8 Hz) activity is closely associated with emotional processing and cognitive control. Asymmetry and connectivity abnormalities in the alpha band (8–14 Hz) are considered potential biomarkers for mood disorders. Meanwhile, the beta band (14–30 Hz), involved in higher-order cognition and motor planning, has also been reported to exhibit changes during depressive states (10). However, research on brain networks related to depressive symptoms in middle school students remains limited. Existing studies predominantly focus on analyses of single frequency bands or specific brain networks, lacking systematic investigations across multiple frequency bands and networks. Furthermore, the relationship between brain activity across different frequency bands and functional connectivity within brain networks has not been fully elucidated in middle school students with depressive symptoms.

Exercise intervention, as a non-pharmacological treatment modality, has gained widespread application in adjunctive depression therapy due to its non-invasive, cost-effective, and easily implementable advantages (9). Empirical research confirms that regular exercise effectively alleviates depressive symptoms. The underlying mechanism may involve regulating brain electrical activity in the prefrontal cortex and limbic system, as well as enhancing alpha-band synchrony (11). Regular Exercise Intervention (REI) and Virtual Reality Exercise Intervention (VR-EI), as two common forms of exercise intervention, may exert distinct effects on brain network functional connectivity (12). REI, through structured physical activity, promotes neurotransmitter release, improves cerebral blood flow, and enhances neuroplasticity, thereby exerting positive regulatory effects on brain network connectivity (13). In contrast, VR-EI integrates virtual reality technology with exercise training. By providing immersive virtual environments, it significantly enhances exercise engagement and interactivity. Preliminary evidence indicates that this multisensory integration intervention can more effectively regulate brain network connectivity in the theta and alpha frequency bands, promote the integration of cognitive-emotional functions (14), and exert a unique regulatory effect on functional connectivity within brain networks (15). However, research on the impact of exercise interventions on brain network functional connectivity in students with depressive symptoms remains in its preliminary stages. Specifically, the precise mechanisms underlying different exercise intervention modalities across distinct frequency bands of brain networks remain unclear.

Based on the above background, this study proposes the following hypothesis: Compared with healthy controls, students with depressive symptoms will exhibit specific patterns of functional connectivity imbalance in the brain networks within the theta [4–8 Hz], alpha [8–14 Hz],and beta [14–30 Hz] bands, specifically manifested as overactivity in the default mode network (DMN),underactivity in the dorsal attention network (DAN), and impaired coordination between networks such as the salience network (SN)and central executive network (CEN). These frequency bands are respectively associated with emotion and cognitive control, resting-state function, and higher-order cognitive-motor planning, comprehensively covering potential neural oscillatory abnormalities underlying depressive symptoms. Furthermore, the study hypothesizes that exercise intervention combined with virtual reality technology (VR-EI), leveraging its multisensory stimulation and immersive experience, will more effectively optimize the aforementioned abnormal network connectivity and activity compared to conventional exercise intervention (REI), accompanied by more significant emotional improvement. By systematically comparing functional connectivity across these three frequency bands and single-network activity levels among different groups, combined with PANAS emotional state evaluations, this study aims to uncover the neural mechanisms underlying depressive symptoms. It further explores the intervention effects of REI versus VR-EI, providing theoretical foundations and practical support for developing precise, personalized early-stage exercise intervention strategies for depression in secondary school students.

## 2 Materials & methods

### 2.1 Experimental subjects

Experiment 1: To investigate brain functional connectivity patterns in resting-state conditions among secondary school students with depressive symptoms, this experiment employed an independent samples t-test. Using G*Power 3.1 software for sample size estimation, with an effect size of 0.80, significance level α = 0.05, and statistical power of 0.95, the minimum total sample size was calculated to be 84 participants. This experiment ultimately recruited 100 secondary school students: 50 in the subclinical depression (ScD) group and 50 in the healthy control (HC) group. Subsequently, two participants in the HC group withdrew midway, reducing the effective sample size to 48. The sample retention rate was 98%, with a total attrition rate of 2%. Therefore, the sample size for both groups met the estimated sample size requirements.

Experiment 2: To explore the interaction effects between different groups and exercise modalities, this experiment employed a 2×3 two-way ANOVA. The required sample size was similarly estimated: with an effect size of 0.40, significance level α = 0.05, and statistical power of 0.80, the minimum total sample size was 64 participants. To balance group sizes and allow for flexibility, 90 participants were recruited (45 in the ScD group and 45 in the HC group). All participants completed the experimental process without dropouts, resulting in a final valid sample size of 90 individuals, which met and exceeded the estimated sample size requirement.

Inclusion criteria required participants aged 13–18 years who underwent depression symptom assessment via the Center for Epidemiologic Studies Depression Scale (CES-D) and Patient Health Questionnaire-9 (*p*HQ-9). Specific screening criteria were CES-D scores ≥ 20 and PHQ-9 scores ≥ 10 (16, 17). All assessment results were reviewed and confirmed by professional counselors from the school counseling center. Participants had no history of alcohol abuse or substance use, no other known medical conditions or developmental abnormalities, and all signed informed consent forms. This study protocol received ethical review approval from the Academic Committee of Shaanxi Normal University (Approval No.: 202516012). Participants were grouped and randomized as follows: First, initial screening was conducted based on CES-D and PHQ-9 scale scores, reviewed by school counselors, and participants were assigned to either the SCD group or the HC group. Subsequently, in Experiment 2, computer-generated random number sequences were used to randomly assign participants within the SCD and HC groups to three intervention conditions (NEC,REI,VR-EI) ensuring 15 participants per condition in each group.

### 2.2 Experimental equipment and assessment tools

This experiment utilizes an immersive virtual reality (VR) power training system based on motion-sensing interaction technology. By integrating a VR engine with smart athletic gear, it establishes an experimental platform with high ecological validity. The system features a PICO 4 professional-grade headset (2160×2160 resolution per eye) and employs free-degree-of-freedom tracking technology to achieve dynamic multi-terrain modeling, encompassing 12 virtual scenarios including urban roads and mountain tracks. Its innovation manifests in three key aspects: (1) A multi-scenario adaptive module dynamically adjusts virtual environment resistance parameters based on user power output, enhancing training realism and adaptability; (2) A psychophysiological dual-dimensional regulation mechanism that dynamically optimizes environmental audio-visual parameters by monitoring heart rate and exercise load in real time, maintaining optimal psychological and physiological states for users; (3) An experimental control hub utilizing wireless screen-casting technology to enable real-time tracking of subjects’ visual focus, ensuring precision and fidelity in intervention implementation.

Primary equipment includes: (1) EEG signal acquisition system: Utilizing a 32-channel Neuroscan EEG system (Brain Vision Recorder) with Curry 8.0 software, operating at a sampling frequency of 1024 Hz. (2) Virtual Reality Motion Platform: An immersive VR power training system built upon the PICO 4 professional-grade headset (monocular resolution 2160×2160),featuring multi-terrain dynamic modeling and adaptive resistance adjustment. (3) Physiological parameter monitoring devices: - ActiGraph GT9-X accelerometer (worn on left wrist)to record physical activity levels. - Polar heart rate monitor (worn below the sternum) for real-time heart rate monitoring.

Psychological assessment tools include: (1) Center for Epidemiologic Studies Depression Scale (CES-D): This 20-item scale assesses the frequency of depressive symptoms over the past week. Total scores range from 0 to 60, with ≥20 indicating significant depressive symptoms. (2) Patient Health Questionnaire-9 (PHQ-9): This 9-item scale is based on DSM-IV depression diagnostic criteria. Total scores range from 0 to 27, with ≥ 10 indicating clinically significant depressive symptoms. (3) Positive and Negative Affect Schedule (PANAS): This scale comprises 20 affective adjectives divided into two dimensions: Positive Affect (PA) and Negative Affect (NA), each with 10 items. Scoring uses a 5-point Likert scale (1 = Not at all, 5 = Extremely), with each dimension ranging from 10 to 50 points. Higher scores indicate more intense emotional experiences.

### 2.3 Intervention protocol

This study examines differences in functional connectivity across three brain networks, heta [4–8 Hz], alpha [8–14 Hz], and beta [14–30 Hz]—between a group of secondary school students with depressive symptoms (ScD) and healthy controls (HC). Two sub-experiments focus on neural mechanisms related to attentional control, self-processing, and emotional perception, as well as how different intervention approaches modulate these mechanisms. Sub-experiment 1 employed independent samples t-tests to compare resting-state brain network characteristics between the ScD group (n=50) and HC group (n=48). Sub-experiment 2 employed a 2 (group: ScD *vs*. HC) × 3 (intervention type: non-exercise control NEC, regular exercise intervention REI, virtual reality exercise intervention VR-EI)factorial design. All interventions occurred in a quiet, temperature-controlled standardized laboratory setting. Each 20-minute session comprised a 3-minute warm-up, 15-minute main intervention, and 2-minute cool-down. Specifically: - NEC group:15 minutes of seated rest without exercise or VR exposure - REI group:15 minutes of stationary cycling training at 50%-80% of individual maximum heart rate (HRmax) -VR-EI group: Same exercise intensity combined with immersive natural landscape VR scenes. The system dynamically adjusted visual scenes and cycling resistance based on real-time power output to enhance training immersion and adaptability. All participants signed informed consent forms prior to the experiment. Data processing strictly adhered to anonymization principles to protect personal privacy.

### 2.4 EEG signal recording and data preprocessing

**Figure 1:**
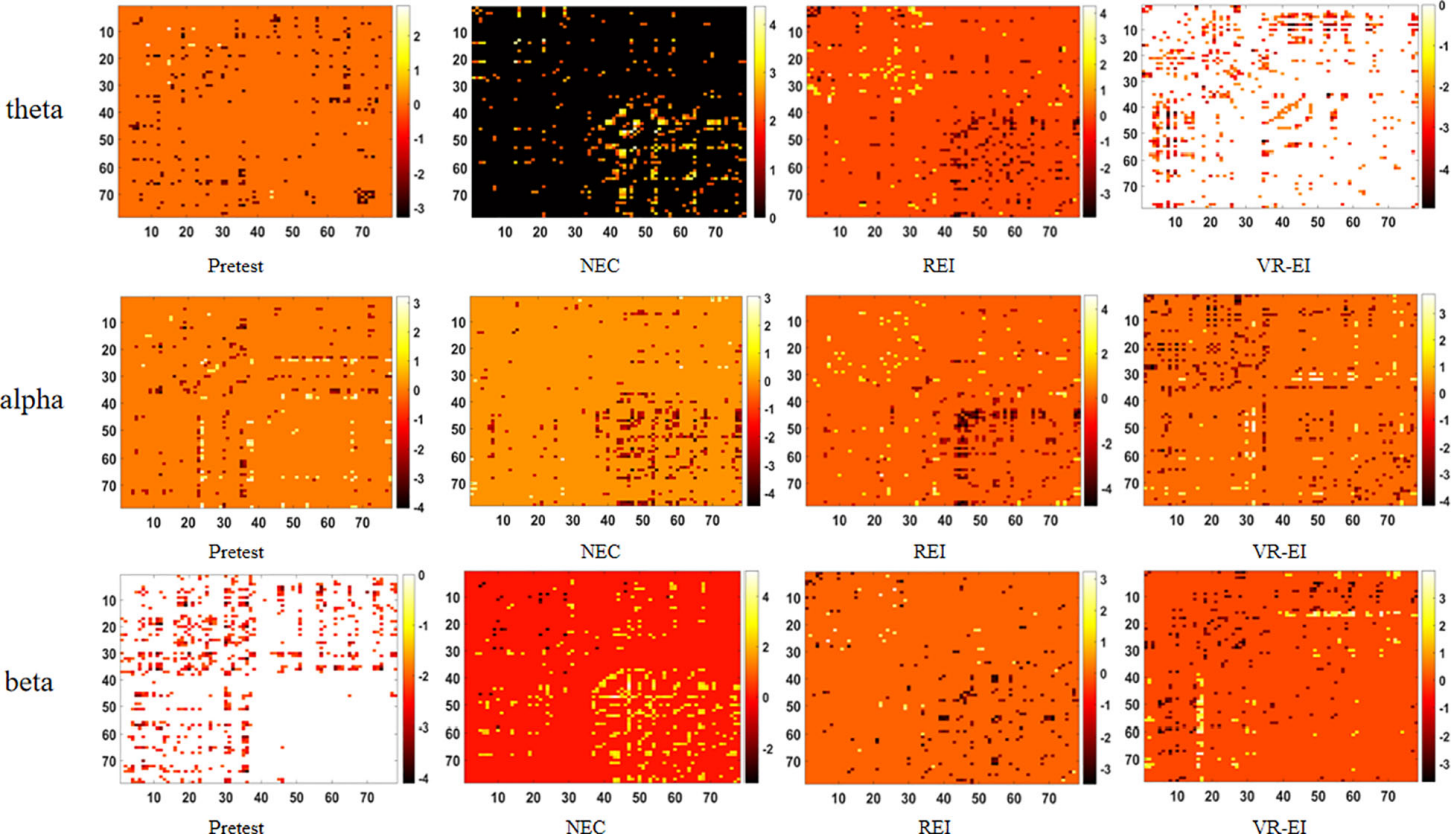
Data acquisition and processing flowchart.

This experiment was conducted in a dimly lit, quiet, soundproof room to minimize external environmental interference during EEG signal acquisition. As shown in the flowchart in **Figure 1**, subjects underwent scalp cleansing prior to the experiment to optimize electrode-scalp contact quality. EEG signals were acquired using a 32-channel EEG recording system (Brain Vision Recorder, Neuroscan, USA) integrated with Curry 8.0 software. Six minutes of resting-state EEG data were recorded from both the student group with depressive symptoms (ScD) and the healthy control group (HC). Electrode-to-scalp contact impedance was strictly maintained below 10 kΩ, with a sampling rate set at 1024 Hz. During the experiment, researchers verbally instructed participants to minimize blinking and head movement to reduce interference from eye movements and electromyographic artifacts, ensuring data quality.

Data preprocessing was performed in MATLAB 2022b using the EEGLAB 2013 toolbox, following these steps: First, irrelevant channels (VEOG/HEOG) were removed. Subsequently, a 1–30 Hz bandpass filter and a 48–52 Hz notch filter were applied to eliminate power supply noise and other high-frequency interference. Reference re-referencing was performed using M1 and M2 electrodes. Separated and removed artifacts such as eye movements and muscle activity using Independent Component Analysis (ICA); discarded outliers exceeding ± 70 μV in signal amplitude; finally, retained only EEG data with durations exceeding 300 seconds for subsequent analysis to ensure data accuracy and reliability.

### 2.5 Network segmentation and static functional connectivity calculation

This study employs the brain atlas template proposed by Schaefer et al. (18), which divides the cerebral cortex into 78 distinct functional regions. This template integrates anatomical structures with functional characteristics, offering high spatial precision and robust neuroscientific interpretability. Building upon this foundation, we further grouped these 78 regions into five core large-scale networks: the sensorimotor network (SMN), dorsal attention network (DAN), salience network (SN), default mode network (DMN), and central executive network (CEN). This framework enables systematic quantification of functional connectivity within brain networks.

**Figure 2:**
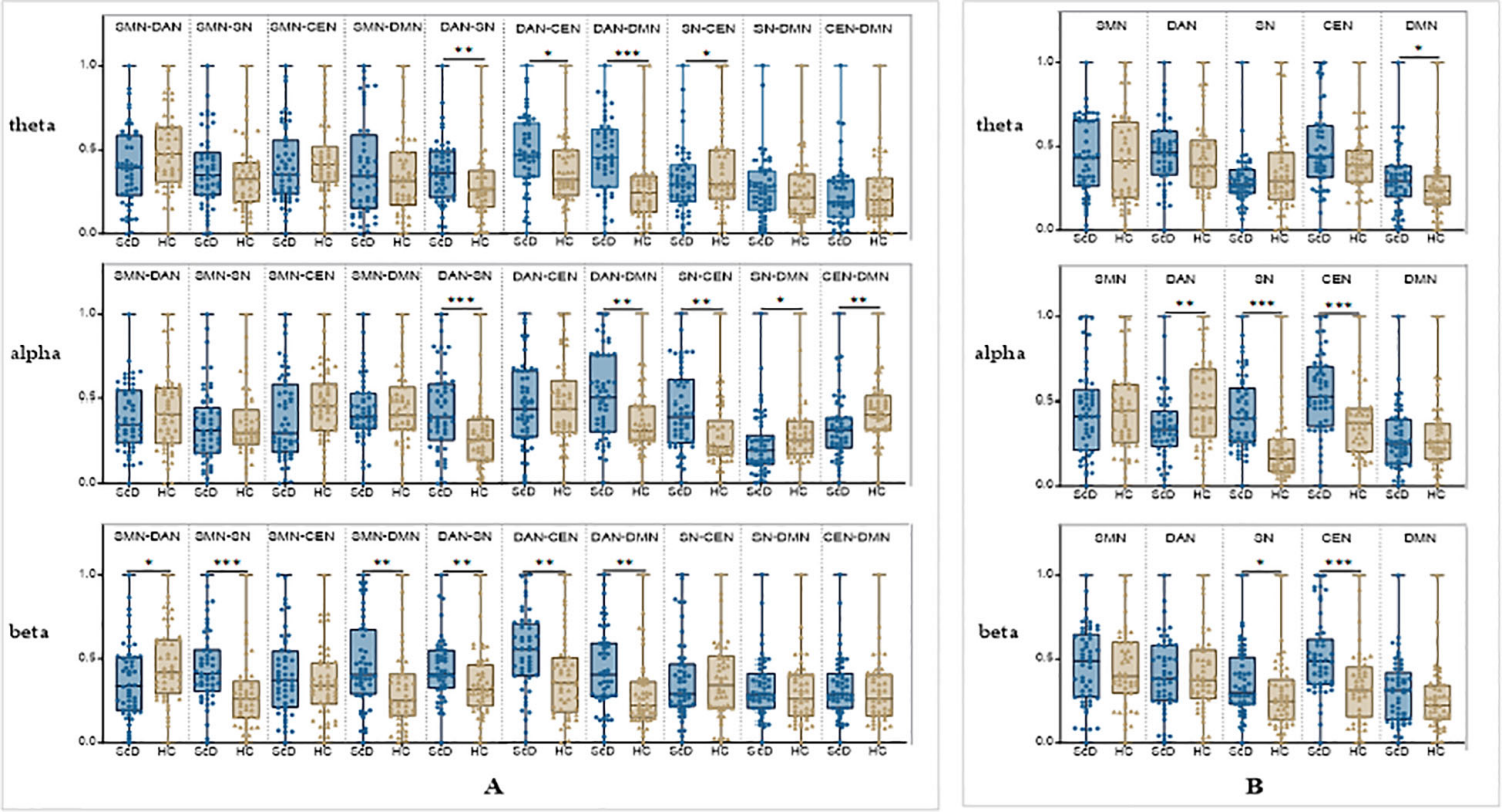
Analysis workflow for resting-state (EEG) brain functional networks. Compared with the HC group, *p < 0.05, **p < 0.01, ***p < 0.001.


To quantify the strength of functional connectivity between networks, the experiment employed Phase Locking Value (PLV) as the primary metric. PLV measures the synchrony of neural activity between different brain regions based on phase information from EEG signals (19) and exhibits strong noise resistance. The specific calculation steps are as follows, with the overall process flow diagram shown in **Figure 2**. First, the instantaneous phase of each brain region is extracted via Hilbert transform. Subsequently, for regions i and j, the phase difference between them is computed within a specified time window T, and its stability is assessed. PLV ranges from [0,1]. Values closer to 1 indicate higher phase synchrony and tighter functional connectivity between brain regions (20), while values closer to 0 suggest lower synchrony and looser functional connections. This method effectively captures the dynamic interaction characteristics between brain networks, providing a reliable quantitative tool for this experiment. Its mathematical expression is:

$$PLV=\frac{1}{T}|\sum_{t=1}^{T}e^{i(\phi_{i}(t)-\phi_{j}(t))}|$$

Here, T denotes the total number of time points within the time window, and represent the instantaneous phases of brain regions i and j at time t, respectively. By calculating the PLV values between networks, we obtain quantitative metrics for functional connectivity strength, providing a reliable data foundation for analyzing interaction patterns between networks and their dynamic changes before and after intervention. The optimized algorithm ensures computational efficiency and result accuracy, enhancing the scientific rigor and interpretability of detailed static network analysis.

### 2.6 Quality control and statistical methods

To ensure the reliability, consistency, and reproducibility of experimental data, this study implemented rigorous quality control measures: all experimenters received standardized training and passed operational consistency assessments; all electronic equipment underwent standardized calibration daily prior to experiments; experiments were conducted between 14:00 - 17:00 daily in the same quiet laboratory to control circadian rhythm effects; All participants received identical standardized instructions and completed experiments under uniform environmental conditions; Two researchers monitored EEG signal quality, heart rate data, and VR device parameters in real-time during experiments to ensure data validity and integrity; EEG artifact removal was performed by two independent researchers, with a third researcher arbitrating disagreements; all EEG data underwent identical preprocessing by a single trained researcher using MATLAB 2022b.

Statistical analyses were uniformly conducted using SPSS 26.0 and MATLAB 2022b, with significance set at *p* < 0.05. Baseline comparisons employed independent samples t-tests; intervention effects were examined using a 2×3 two-way mixed ANOVA to assess main effects and interaction effects between group (ScD *vs*. HC)and intervention condition (NEC, REI,VR-EI). When interaction effects were significant, Bonferroni *post-hoc* tests were conducted. Network-level multiple comparisons additionally utilized NBS correction. These measures collectively ensured standardized procedures and high-quality reproducibility throughout experimental operations, data collection, and analysis.

## 3 Results

### 3.1 Comparison of participant demographics and scale scores

**Table 1** presents characteristics of participants’ depressive symptom levels. p-values were calculated using t-tests or chi-square tests based on the specific variable type. Results indicate significant differences in CES-D and PHQ-9 scores between the Subclinical Depression (ScD) group and the Healthy Controls (HC) group (*p* < 0.001).

**Table 1:** Comparison of demographic and scale scores between ScD and HC groups (Mean ± SD).

Variable	ScD group (n = 50)	HC group (n = 48)	*t*	*p*
Age	14.12 ± 0.773	14.19 ± 0.734	-0.443	0.659
Gender (Male/Female)	24/26	23/25	-0.008	0.993
CES-D Score	27.90 ± 7.864	6.33 ± 6.131	2.790	0.000
PHQ-9 Score	11.94 ± 2.161	2.77 ± 2.684	27.401	0.000

**Table 2** presents baseline measurements from the Positive and Negative Affect Schedule (*p*ANAS) prior to intervention, comparing differences in positive affect (*p*A) and negative affect (NA) between ScD and HC groups. The PANAS scale comprises 20 items, with Positive Affect (*p*A) consisting of 10 items (e.g., “excitement,” “enthusiasm,” ‘pride’) and Negative Affect (NA) comprising 10 items (e.g., “nervousness,” “fear,” “guilt”). Each item is rated on a 5-point Likert scale (1 = not at all, 5 = extremely), with total scores ranging from 10 to 50. Higher scores indicate stronger emotional experiences. Results showed that the PA score in the ScD group (M = 19.260 ± 2.813) was significantly lower than that in the HC group (M = 30.083 ± 1.867, t = -22.351, *p* < 0.001), indicating significantly reduced positive emotional experiences in the ScD group. Concurrently, the ScD group’s NA score (M = 32.620 ± 4.280) was significantly higher than that of the HC group (M = 14.583 ± 1.674, t = 27.257, *p* < 0.001), reflecting markedly heightened negative emotional experiences in the ScD group. Negative affect scores exceeding 25 (*p*articularly in the 30–50 range) indicate intense negative emotions potentially linked to anxiety, stress, or emotional distress (21). The ScD group’s NA scores surpassing 30 further underscore the need for mental health attention, especially in clinical screening for anxiety or depressive disorders (22).

**Table 2:** Comparison of positive and negative affect (*p*ANAS) scores between ScD and HC groups.

Variable	ScD	HC	*t*	*p*
PA	19.260 ± 2.813	30.083 ± 1.867	-22.351	0.000
NA	32.620 ± 4.280	14.583 ± 1.674	27.257	0.000

**Table 3** presents the measurement results of the Positive and Negative Emotion Scale following the intervention. In the Non-Exercise Control (NEC) group, the Regular Exercise Intervention (REI) group, and the Virtual Reality Exercise Intervention (VR-EI) group, ScD group showed significant increases in positive emotion from NEC (M = 15.200 ± 1.146) to REI (M = 24.600 ± 1.682) and VR-EI (M = 27.267 ± 1.981), with VR-EI demonstrating superior effects compared to REI (*p* < 0.05); Negative emotions decreased significantly from NEC (M = 28.667 ± 2.093) to REI (M = 21.267 ± 3.788) and VR-EI (M = 15.933 ± 1.907), with VR-EI showing the most pronounced effect (*p* < 0.001). PA and NA also improved in the HC group, but to a lesser extent. Interaction effect analysis revealed that both group and condition significantly influenced positive emotion (F = 44.359, *p* = 0.000, *η*² = 0.613) and negative emotion (F = 38.002, *p* = 0.000, *η*² = 0.576). Overall, both REI and VR-EI interventions significantly improved the emotions of ScD patients, with VR-EI demonstrating superior efficacy.

**Table 3:** Emotional changes and interaction effects analysis of PANAS under different intervention conditions.

Variable	Group	ScD	HC	Interaction effect		
				F	*p*	*η* ^2^
PA	NEC	15.200 ± 1.146	17.533 ± 2.167^**^	44.359	0.000	0.613
	REI	24.600 ± 1.682^###^	18.867 ± 2.722^**^			
	VR-EI	27.267 ± 1.981^###&^	19.200 ± 3.005^***^			
NA	NEC	28.667 ± 2.093	18.467 ± 2.532^***^	38.002	0.000	0.576
	REI	21.267 ± 3.788^###^	18.600 ± 3.019*			
	VR-EI	15.933 ± 1.907^###&&&^	18.000 ± 2.449*			

Compared with the HC group, *p < 0.05, **p < 0.01, ***p < 0.001; Compared with the REI group, ###*p* < 0.001; Compared with the VR-EI group, &*p* < 0.05, &&&*p* < 0.001.

### 3.2 Analysis of differences in static brain functional connectivity between depressed adolescents and healthy controls

**Figure 3:**
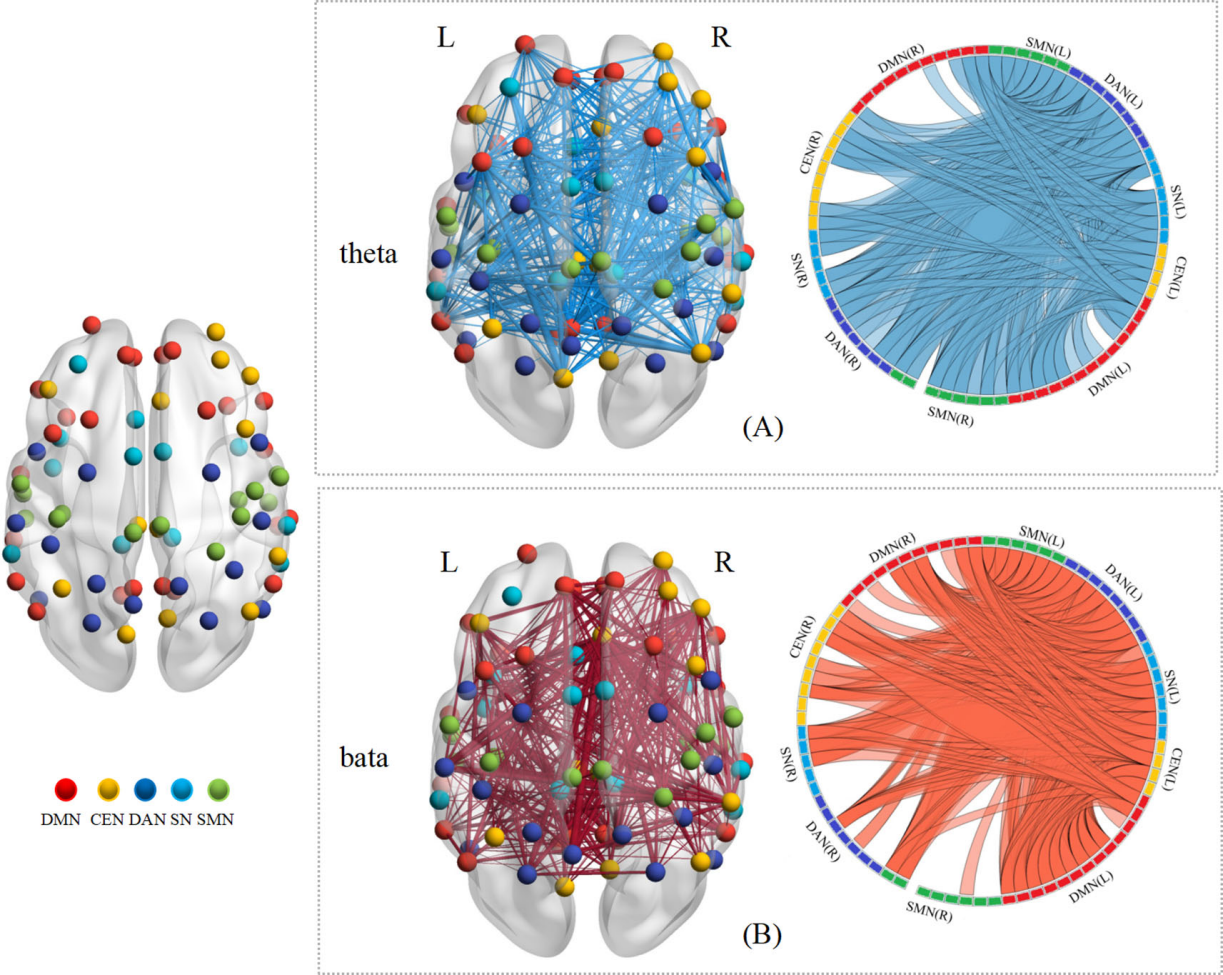
Comparison of functional connectivity differences between ScD and HC in the theta, alpha, and beta frequency bands under different intervention conditions.

This study employed an independent samples t-test combined with network-based statistics (NBS correction parameters: Edge *p* < 0.05, component *p<* 0.05, with 2000 permutations) to systematically compare functional connectivity differences in the ScD group and HC group across baseline (*p*retest) and three intervention conditions (NEC, REI, VR - EI) within the theta (4–8 Hz), alpha (8–14 Hz), and beta (14–30 Hz) frequency bands. **Figure 3** presents the 78×78 functional connectivity matrix across 78 brain regions, analyzing connection strengths within the default mode network (DMN), dorsal attention network (DAN), salience network (SN), central executive network (CEN), and sensorimotor network (SMN). This aims to reveal differences in attention regulation, self-processing, and emotional perception neural mechanisms between ScD and HC groups, along with intervention effects.

**Figure 4:**
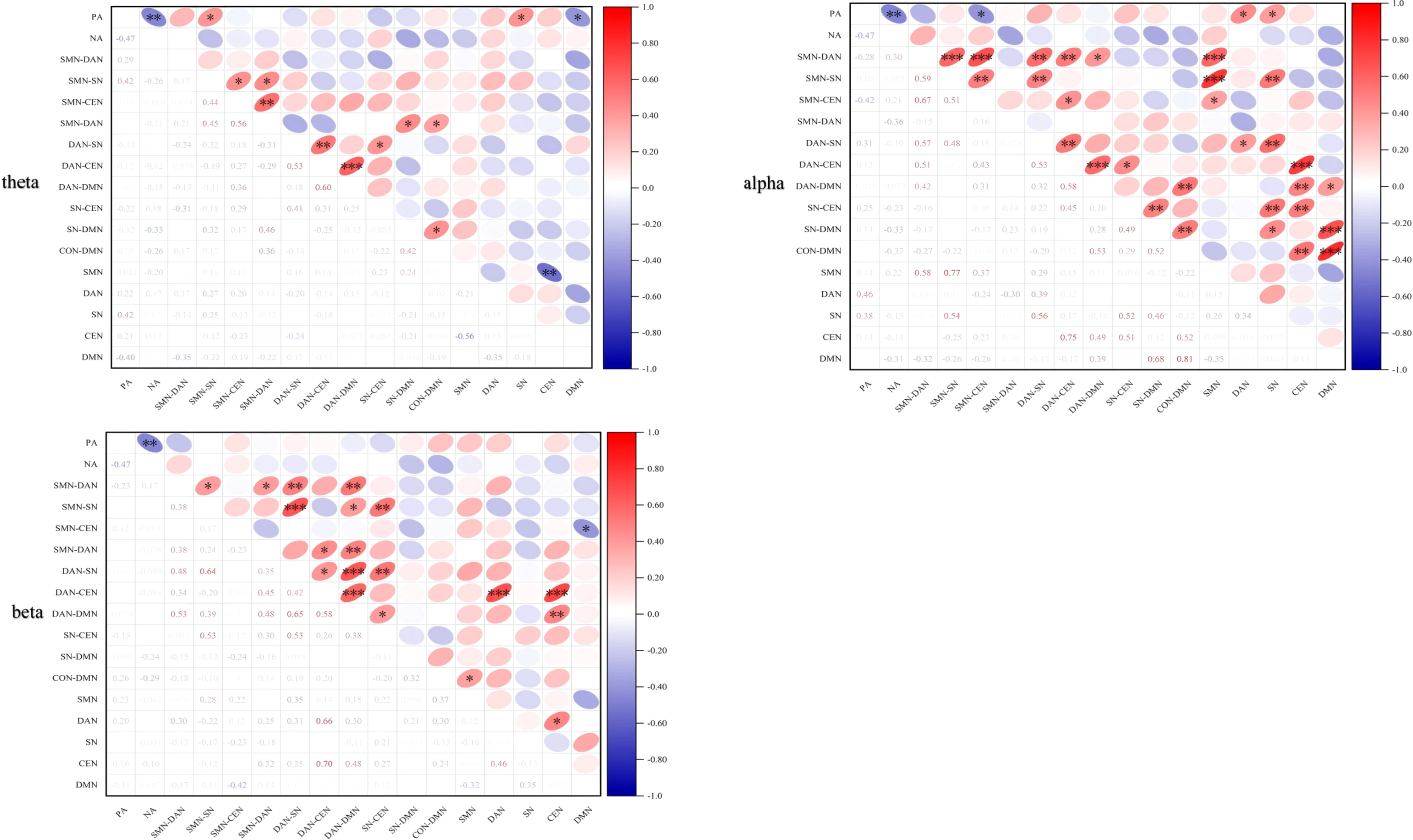
Visualization of differences in functional connectivity within brain networks. *p < 0.05, **p < 0.01, ***p < 0.001.

Research findings indicate that in the θ band, functional connectivity strength in the ScD group was significantly higher than in the HC group following VR - EI intervention (*p* = 0.011). In the α band, no significant differences were observed between the two groups, suggesting lower sensitivity of this band to ScD neural features or insufficient intervention effects. In the β band, during the pre-test state, functional connectivity strength in the ScD group was significantly lower than that in the HC group (*p* = 0.0499). Detailed results are presented in **Figure 4** (visualization of brain network functional connectivity). In the figure, red, yellow, dark blue, light blue, and green represent the Default Mode Network (DMN), Central Executive Network (CEN), Dorsal Attention Network (DAN), Salience Network (SN), and Somatomotor Network (SMN), respectively. Blue indicates enhanced connectivity strength, while red indicates weakened connectivity strength. **Figure 4A** demonstrates that in the θ band, the ScD group exhibited significantly enhanced connectivity across all networks following VR - EI intervention. **Figure 4B** indicates that in the β band during the pre-test state, the ScD group showed significantly reduced connectivity across all networks. These findings further validate the presence of significant abnormalities in brain functional connectivity at higher frequency bands in ScD, providing crucial scientific evidence for elucidating the neural mechanisms of ScD and evaluating intervention efficacy.

### 3.3 Baseline differences in resting-state brain networks between depressed students and healthy controls prior to intervention

**Figure 5:**
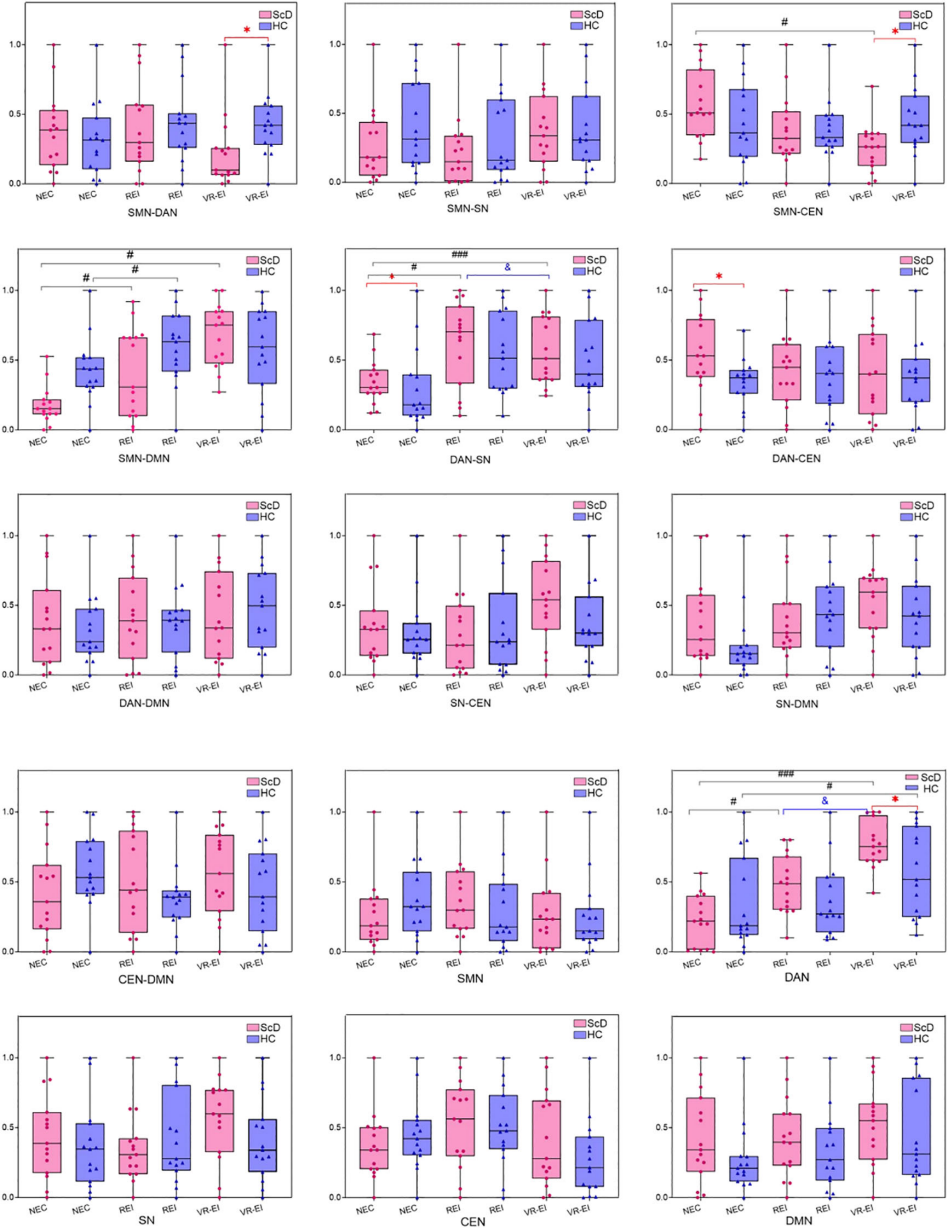
Changes in brain functional network connectivity strength in ScD students at rest. [**(A)**: Inter-network connectivity strength]. **(B)**: Intra-network connectivity strength]. Compared with the HC group, *p < 0.05; Compared with the REI group, #p < 0.05, ###p < 0.001; Compared with the VR-EI group, &p < 0.05.

This study systematically compared functional connectivity strength and single-network activity levels across the DMN, DAN, SN, CEN, and SMN networks in the theta, alpha, and beta frequency bands between ScD and HC groups using independent samples t-tests based on EEG resting-state data. **Figure 5** results indicate that the ScD group exhibited significant characteristic differences in composite metrics across multiple key brain networks.

In the theta band, ScD exhibited significantly enhanced functional connectivity between networks such as DAN-SN, DAN-CEN, and DAN-DMN (*p* = 0.007, 0.013, and 0.000, respectively), while connectivity between SN and CEN was significantly weakened (*p* = 0.028). Concurrently, the DMN’s single-network activity level also significantly increased (*p* = 0.025), reflecting a potential enhancement in self-processing tendencies. No significant differences were observed in the remaining combination metrics.

In the alpha band, ScD similarly exhibited enhanced connectivity in DAN-SN, DAN-DMN, and SN-CEN (*p* = 0.001, 0.003, 0.001), while SN-DMN and CEN-DMN connectivity significantly decreased (*p* = 0.014 and 0.004). DAN’s alpha activity level was significantly lower than HC (*p* = 0.005), while SN and CEN activity was significantly elevated (*p* < 0.001), suggesting that while the external attention system was downregulated, the salience system was in a state of overactivation.

In the beta band, ScD showed enhanced connectivity in SMN-SN, SMN-DMN, DAN-SN, DAN-CEN, and DAN-DMN connections, while exhibiting weakened connectivity in the SMN-DAN connection. Second, although the SN-CEN, SN-DMN, and CEN-DMN combination connections did not reach significant differences, the activity levels of the two single networks, SN and CEN, were significantly elevated in ScD (*p* = 0.027, *p* = 0.000), possibly reflecting overload at the levels of emotional arousal and cognitive control.

### 3.4 Effects of different intervention strategies on multi-band resting-state brain network activity in depressed students

**Figure 6:**
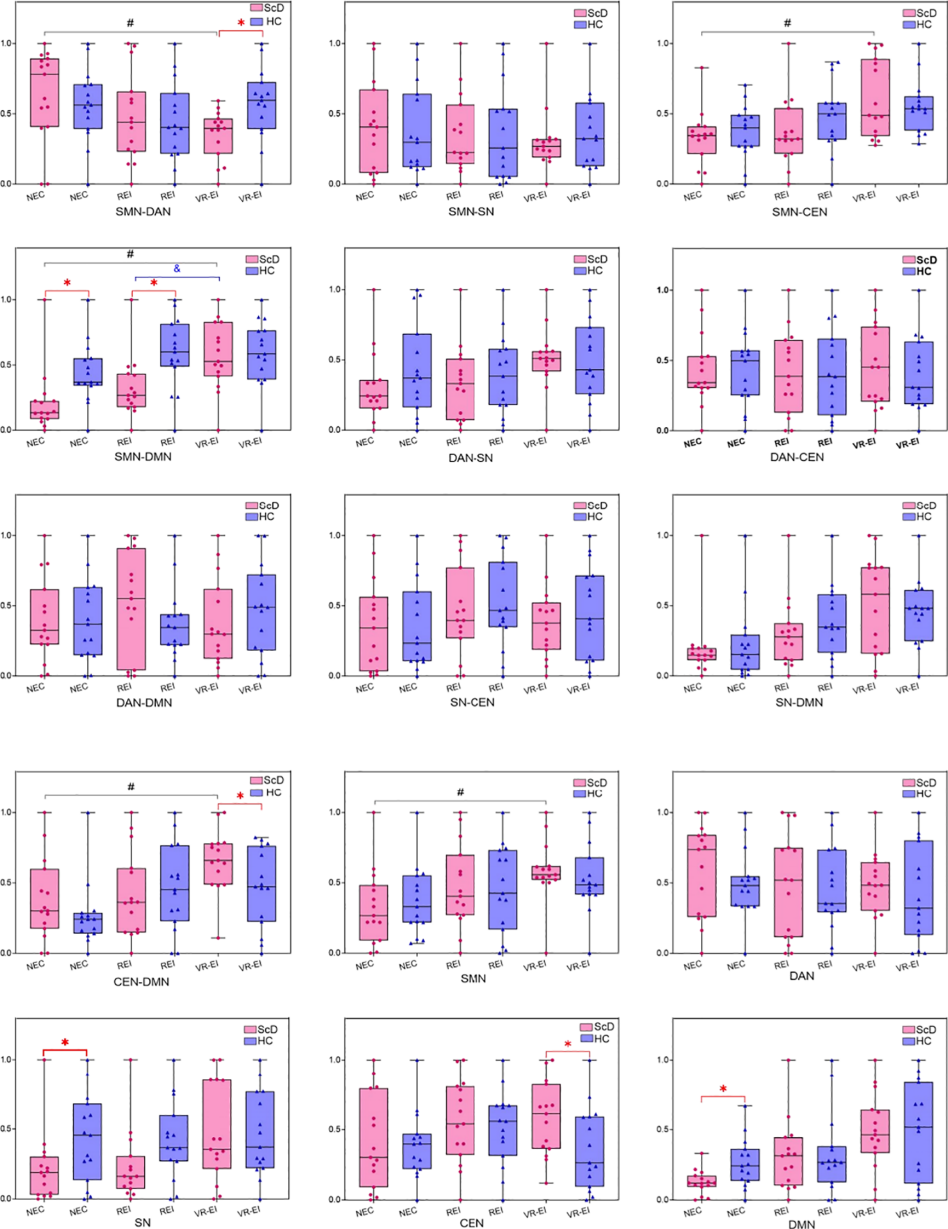
Comparison of intra - and inter-network activity levels in ScD and HC groups across different intervention modalities in the θ band. (Compared with the HC group, *p < 0.05; Compared with the REI group, #*p* < 0.05 Compared with the VR-EI group, &*p* < 0.05.

**Figure 7:**
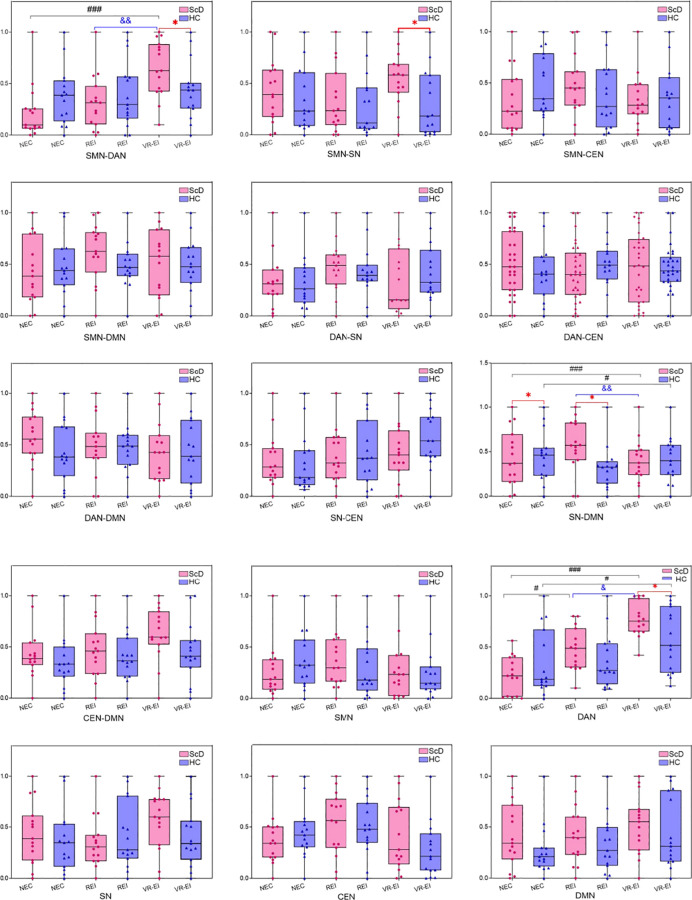
Comparison of intra - and inter-network activity levels in ScD and HC groups across different intervention modalities in the alpha band. (Compared with the HC group, *p < 0.05, **p < 0.01, ***p < 0.001 Compared with the REI group, #*p* < 0.05, ###*p* < 0.01, ###*p* < 0.001; Compared with the VR-EI group, &*p* < 0.05, &&*p* < 0.01, &&&*p* < 0.001).

**Figure 8:**
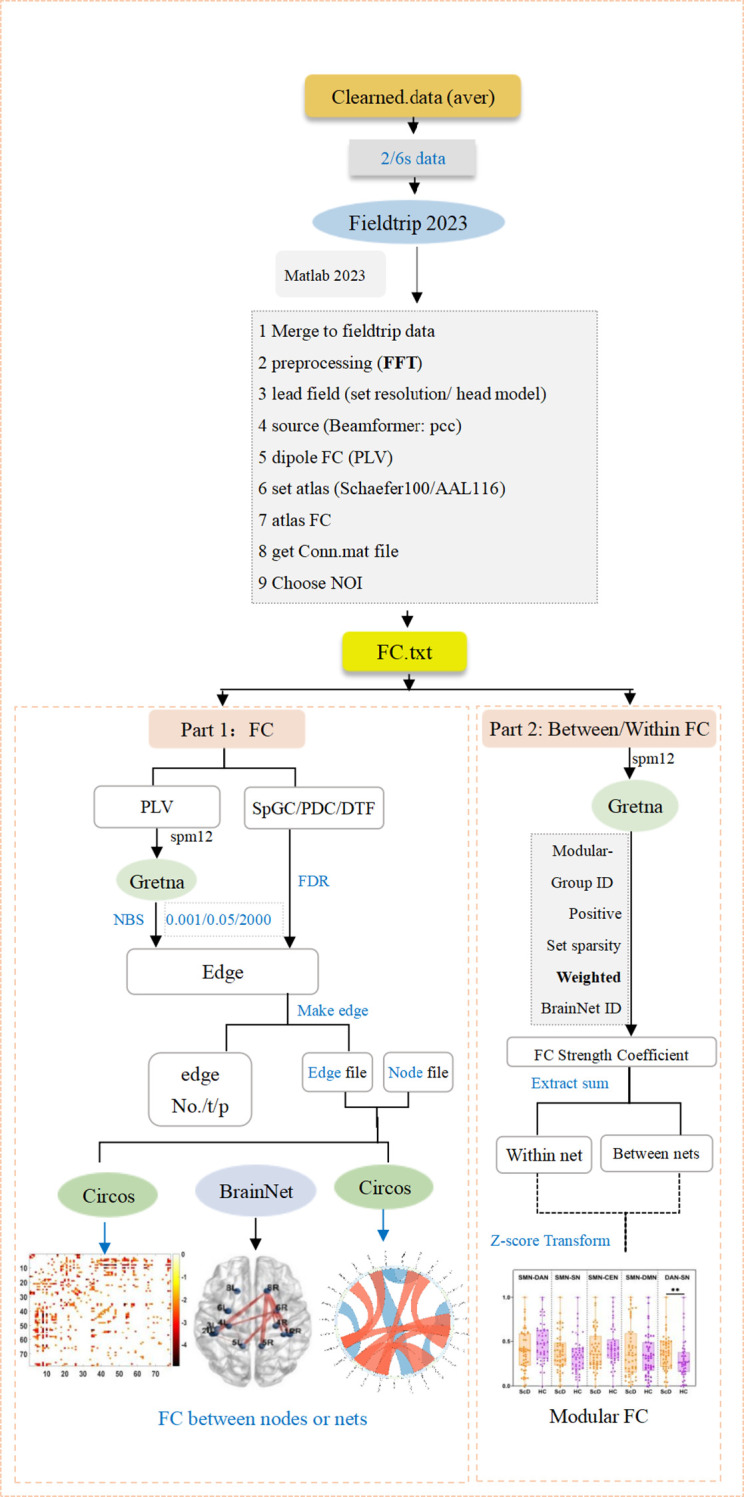
Comparison of intra - and inter-network activity levels between ScD and HC groups under different intervention methods in the beta band. Compared with the HC group.

This study systematically evaluated differences in functional connectivity strength and single-network activity levels across the DMN, DAN, SN, CEN, and SMN networks in ScD and HC groups across three frequency bands (theta, alpha, beta) and three intervention conditions (NEC, REI, VR-EI) using EEG resting-state data and two-way ANOVA. Analysis results (see **Figures 6**–**8**) revealed significant characteristic differences in the ScD group across multiple key brain networks’ composite metrics, suggesting potential abnormalities in neural mechanisms related to attentional regulation, self-processing, and emotional perception.

In the θ band, significant interaction effects were observed for SMN-CEN (F(0.441, 3.458) = 3.574, *p* = 0.035, *η*² = 0.113) and DAN-SN (F(0.534, 3.653) = 4.096, *p* = 0.023, *η*² = 0.128). Under VR-EI intervention, the ScD group exhibited significantly lower SMN-CEN connectivity than the HC group (*p* < 0.01), indicating deficits in motor-cognitive control integration. Under NEC conditions, the ScD group showed significantly lower SMN-DAN connectivity than the HC group (*p* < 0.01), suggesting impaired neural coordination between motor and attentional integration, which may affect their ability to allocate attentional and motor resources effectively during complex tasks. Both SMN-DMN and DAN-SN connectivity in the ScD group significantly improved after REI and VR-EI interventions compared to NEC (*p* < 0.05), with VR-EI outperforming RET (*p* < 0.05). This demonstrates VR-EI’s significant efficacy in enhancing SMN-DMN connectivity and attention network function.

DAN activity (F(0.415, 3.308) = 3.516, *p* = 0.042, *η*² = 0.112) was significantly higher in the VR-EI intervention group than in the HC group (*p* < 0.01). The ScD group showed significantly greater VR-EI intervention effects than both NEC and REI (*p* < 0.001), indicating that VR-EI enhances attentional network activity. Following VR-EI intervention in the ScD group, SN-DMN and SN-CEN connectivity values increased compared to NEC (0.530 ± 0.262 *vs*. 0.374 ± 0.314; 0.535 ± 0.303 *vs*. 0.362 ± 0.286), though no significant interaction effects were observed, suggesting a potential improvement trend.

In the alpha band, significant interaction effects were observed for SMN-DAN (F(0.589, 3.671) = 4.494, *p* = 0.016, *η*² = 0.138) and DMN activity (F(0.598, 4.775) = 3.507, *p* = 0.041, *η*² = 0.111). Under VR-EI intervention, the ScD group exhibited significantly higher SMN-DAN connectivity strength (M = 0.650 ± 0.274) than the HC group (M = 0.443 ± 0.285, *p* < 0.01). Both REI (M = 0.314 ± 0.268) and VR-EI (M = 0.650 ± 0.274) interventions in the ScD group significantly improved compared to NEC (M = 0.223 ± 0.259), with VR-EI outperforming REI, indicating VR-EI effectively enhances motor-attention integration. DMN activity in the ScD group showed significantly enhanced VR-EI (M = 0.564 ± 0.239) compared to NEC (M = 0.314 ± 0.252) (*p* < 0.05), suggesting VR-EI promotes introspective cognitive function. SN-DMN connectivity in the ScD group was significantly lower than in the HC group under both NEC and REI conditions (*p* < 0.001), with significant improvement after VR-EI intervention (*p* < 0.001), demonstrating VR-EI’s role in coordinating emotional salience and default mode. SMN-SN and DAN activity showed significantly enhanced connectivity strength in the ScD group under VR-EI intervention (*p* < 0.05), while connections such as SMN-CEN and DAN-CEN exhibited no significant interaction effects and remained stable, indicating the targeted influence of VR-EI intervention on specific networks.

In the beta band, no significant interaction effects were observed. However, *post hoc* analysis revealed that VR-EI intervention significantly enhanced SMN, SMN-DMN, and CEN-DMN activity in the ScD group (*p* < 0.05). SMN-DMN activity was significantly lower than the HC group under NEC and REI conditions (*p* < 0.05), but significantly higher than NEC and REI after VR-EI intervention (*p* < 0.05), suggesting VR-EI improves connectivity between motor and default modes. CEN-DMN and CEN activity were significantly higher than the HC group under VR-EI conditions (*p* < 0.05). CEN-DMN and SMN activity significantly increased under VR-EI intervention (*p* < 0.05), while SMN-DMN activity was significantly lower than NEC after VR-EI intervention (*p* < 0.05), potentially reflecting the impact of overstimulation in the high-frequency band. SN and DMN activity in the ScD group were significantly lower than the HC group under NEC conditions (*p* < 0.05). Following VR-EI intervention (M = 0.487 ± 0.273), DMN activity in the ScD group was significantly higher than under NEC (M = 0.126 ± 0.085, *p* < 0.05), indicating that VR-EI exerts a certain degree of improvement on emotional and cognitive networks in the high-frequency band.

### 3.5 Association between brain network changes across different frequency bands and emotional improvement under virtual reality exercise intervention

**Figure 9:**
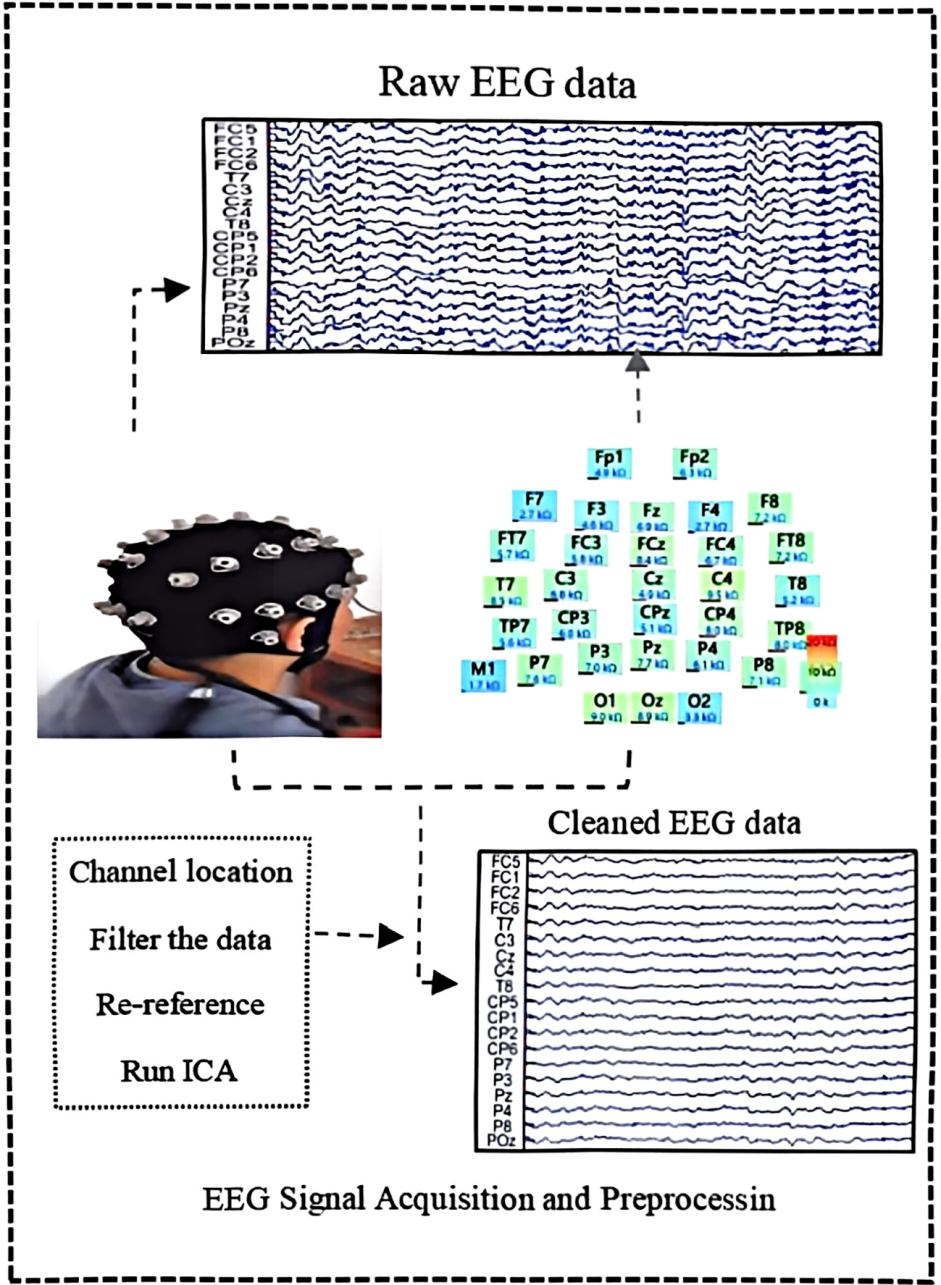
Heatmap showing correlations between brain network metrics in the θ, α, and β bands and emotional improvement in the ScD group following virtual reality exercise intervention.

This study further explored the neurobehavioral mechanisms underlying VR exercise intervention’s improvement of depressive symptoms through Pearson correlation analysis. In the VR-EI group, we calculated the correlation between post-intervention changes in brain network metrics and changes in positive emotion. Results revealed that in the θ band (**Figure 9**), enhanced activity in the dorsal attention network (r = 0.46, *p* = 0.042) and the salience network (r = 0.38, *p* = 0.038) were significantly positively correlated with positive emotion enhancement. Conversely, increased connectivity between the sensorimotor network and the central executive network showed a significant negative correlation with positive emotion enhancement (r = -0.42, *p* = 0.029). In the alpha band, enhanced sensorimotor-salience network connectivity (r = 0.42, *p* = 0.031), increased salience network activity (r = 0.42, *p* = 0.028), and heightened default mode network activity (r = 0.40, *p* = 0.035) all showed significant positive correlations with positive emotion elevation.

## 4 Discussion

### 4.1 Functional imbalance in brain networks of students with depressive symptoms

Compared with healthy controls, students with depressive symptoms exhibited significant neurofunctional imbalances in brain network connectivity and single-network activity across the θ, α, and β frequency bands. This reveals specific neural abnormalities in attention, emotion, and self-referential functions, providing crucial insights for understanding the neural mechanisms of depressive symptoms and designing interventions (23). In the θ band, the dorsal attention network (DAN) in the ScD group showed significantly enhanced functional connectivity with the salience network (SN), central executive network (CEN), and default mode network (DMN) (*p* = 0.007, 0.013, 0.000, respectively), while SN-CEN connectivity was significantly weakened (*p* = 0.028). DMN activity levels were significantly elevated (*p* = 0.025), reflecting excessive activation of self-referential and ruminative thinking. This aligns with prior findings of DMN hyperactivity in depressed patients (24). These multi-frequency imbalance patterns align closely with recent adolescent EEG studies, further confirming that subclinical depression exhibits similar switching impairments across the three-network model (DMN-SN-CEN) observed in clinical depression (25). Enhanced DAN-SN and DAN-DMN connectivity may lead to excessive allocation of attentional resources toward emotional salience or introspective processes, limiting task-directed cognitive processing. Conversely, weakened SN-CEN connectivity may impair frontostriatal coordination, affecting emotional regulation capacity (26). In the alpha band, enhanced DAN-SN, DAN-DMN, and SN-CEN connectivity (*p* = 0.001, 0.003, 0.001, respectively), while SN-DMN and CEN-DMN connectivity decreased (*p* = 0.014, *p* = 0.004, respectively). DAN activity decreased (*p* = 0.005), while SN and CEN activity increased (*p* < 0.001). These findings indicate that neural oscillations in the alpha band reflect downregulation of the external attentional system (DAN) and excessive activation of the emotional salience systems (SN and CEN) (22), potentially leading to impaired coordination of emotion regulation and self-referential processing. This may correlate with negative bias and attentional deficits observed in depressive symptoms (27). In the β band, enhanced connectivity was observed between SMN-SN, SMN-DMN, and DAN-SN, while SMN-DAN connectivity weakened (*p* < 0.05). Elevated SN and CEN activity (*p* = 0.027 and *p* = 0.000, respectively) suggested impaired integration of motor and attentional processes, along with overload in emotional and cognitive control systems (28). This aligns with the β band’s role in higher-order cognitive processing and high-frequency neural oscillations, potentially reflecting impaired behavioral initiative and emotional regulation during complex tasks in the ScD group (29). These multi-band brain network imbalances indicate that neural mechanisms in the ScD group involve abnormal interactions between attention allocation, emotional salience, and self-referential functions, providing neural targets for precision interventions (30). Future research should further explore the dynamic interaction mechanisms across different frequency bands and validate the spatial specificity of EEG findings using functional magnetic resonance imaging (fMRI).

### 4.2 Effects of exercise interventions on brain network function

This study employed a 2 (group: ScD *vs* HC) × 3 (intervention: NEC, REI, VR-EI) factorial design to systematically investigate the effects of NEC and VR-EI interventions on brain network functional connectivity and single-network activity in ScD. Results indicated that VR-EI intervention demonstrated significantly superior efficacy to REI in the θ and α bands, with complex but locally improved effects in the β band. In the θ band, VR-EI significantly enhanced SMN-DMN and DAN-SN connectivity (*p* < 0.05), with DAN activity markedly higher than the HC group (*p* < 0.01) and superior to NEC and REI (*p* < 0.001). However, SMN-CEN and SMN-DAN connectivity remained below HC levels (*p* < 0.01). Although SN-DMN and SN-CEN connectivity increased (0.530 ± 0.262 *vs*. 0.374 ± 0.314; 0.535 ± 0.303 *vs*. 0.362 ± 0.286), no significant interaction effects were observed. This suggests VR-EI promotes neural coordination between motor and self-reference, attention and emotional salience through immersive multisensory stimulation, potentially enhancing attentional regulation and emotional integration by activating the prefrontal cortex and limbic system (31). Persistent deficits in SMN-CEN and SMN-DAN connectivity indicate challenges in motor-cognitive control and attentional integration, necessitating further optimization of intervention design. In the alpha band, VR-EI significantly enhanced SMN-DAN (F(0.589, 3.671) = 4.494, *p* = 0.016, *η*² = 0.138), SN-DMN (*p* < 0.001), SMN-SN connectivity, and DMN/DAN activity (*p* < 0.05), outperforming REI. Enhanced SMN-DAN connectivity improved motor-attention integration, while strengthened SN-DMN connectivity facilitated coordination between emotional salience and self-referential processing. Increased DMN activity may mitigate depression-related rumination (32). The stability of SMN-CEN and DAN-CEN connectivity suggests limited modulatory effects of VR-EI on these networks, potentially due to insufficient intervention duration or stimulus specificity. In the β band, VR-EI enhanced SMN, SMN-DMN, CEN-DMN, and SMN-CEN connectivity in the ScD group (*p* < 0.05), but SMN-DAN connectivity remained below NEC levels (*p* < 0.05). Interestingly, this partially contradicts findings from Du et al. (33), potentially due to excessive activation from the high-intensity stimulation selected in this study, which may have constrained integration between motor functions and attention/cognitive control. DMN activity increased significantly from NEC levels below HC group (*p* < 0.05) to post-VR-EI intervention (*p* < 0.05), supporting enhanced introspective cognition (34). SN activity showed no significant improvement, limiting the regulatory effect on emotional salience. Overall, VR-EI significantly optimized brain network function across multimodal neural pathways in the θ and α bands (35), though stimulation intensity in the β band requires optimization to avoid overactivation. Future studies should extend intervention duration, explore targeted effects of different VR-EI tasks on specific networks, and validate the causal relationship between improved functional connectivity and emotional changes using behavioral data.

### 4.3 Differences in intervention modalities and their potential mechanisms

Compared to REI, VR-EI intervention demonstrated significant advantages in alleviating brain network dysfunctions associated with ScD, particularly in the theta and alpha frequency bands. It significantly outperformed REI in improving SMN-DAN, SMN-DMN, DAN-SN, and SN-DMN connectivity, as well as DMN and DAN activity (*p* < 0.05). VR-EI activates multimodal neural pathways linking the prefrontal cortex (involving DAN and CEN) with the limbic system (involving SN and DMN) through visual, auditory, and motor feedback, thereby enhancing functional connectivity between networks. This may regulate emotional and cognitive functions via the dopaminergic system (36, 37). Significant responses in the θ and α bands align with these frequencies’ critical roles in attention, emotion, and introspective cognition (37), consistent with Nuechterlein’s findings. VR-EI’s immersive experience improves motor-attention integration by enhancing SMN-DAN connectivity, optimizes coordination of emotional salience and self-referentiality through SN-DMN connections, and mitigates rumination and attention deficits by boosting DMN and DAN activity. REI primarily elevates brain-derived neurotrophic factor (BDNF) levels through physical activity (38, 39), promoting neuroplasticity. However, lacking multisensory stimulation, it exhibits weaker regulation of inter-network connectivity, showing only limited improvements (*p* < 0.05) in select networks (e.g., SMN-DMN). In the β band, VR-EI significantly improved SMN-DMN, SMN-CEN, and CEN-DMN connectivity (*p* < 0.05), promoting integration of motor, self-referential, cognitive control, and introspective functions. However, SMN-DMN connectivity weakened (*p* < 0.05), leading to imbalanced neural resource allocation. This correlates with the β band’s involvement in higher-order cognitive processing and high-frequency neural oscillations (40), suggesting VR-EI tasks require optimized stimulation intensity to avoid overactivation. In contrast, Real-Environment Interaction (REI) exhibited weaker effects in the β band and failed to significantly enhance high-frequency network connectivity. VR-EI activates multimodal neural pathways through multisensory stimulation and immersive experiences, outperforming REI across multiple frequency bands to enhance neural coordination. However, the complex effects in the beta band indicate the need for further refinement of stimulus parameters. Future research should explore the targeted effects of specific VR-EI task components (e.g., visual complexity, motor intensity) on different frequency bands. Integrating neuroimaging techniques (e.g., fMRI) to validate the role of the dopamine system in regulating the salience network will optimize intervention efficacy (41).

### 4.4 Individual differences and personalized intervention strategies

Individual differences significantly influence adolescents’ response to exercise interventions for depressive symptoms, making personalized strategies crucial for optimizing treatment outcomes. Research indicates that individuals with hyperactive DMN benefit more from VR-EI intervention, which effectively regulates introspective cognition and alleviates rumination by enhancing SMN-DMN and SN-DMN connectivity (*p* < 0.05). Individuals with lower SMN-DAN or SN-DMN connectivity at pre-test baseline demonstrated heightened sensitivity to VR-EI improvements: enhanced SMN-DAN connectivity improved motor-attention integration (*p* < 0.01), while improved SN-DMN connectivity promoted emotional regulation (*p* < 0.001); Individuals with pronounced attention deficits improved attentional allocation through VR-EI by enhancing SMN-DAN connectivity and DAN activity (*p* < 0.05); those with strong introspective tendencies exhibited significantly heightened DMN activity during VR-EI (*p* < 0.01). These findings indicate that baseline brain network states and cognitive-emotional styles shape intervention outcomes, consistent with personalized neuromodulation theory. To optimize efficacy, tailored interventions should target specific regions based on baseline EEG characteristics (e.g., DMN/DAN activity levels), prioritizing VR-EI protocols that enhance SMN-DAN, SMN-DAN, DAN-SN, and SN-DMN connections in the θ and α bands. Low-frequency VR-EI tasks (e.g., slow-paced movement, guided meditation) should be designed to maximize integration of attention, emotion, and introspective cognition. In the β band, SMN-DAN connectivity requires optimization by controlling stimulus intensity (e.g., reducing complexity of visual or motor feedback) to maintain high-frequency neural oscillation balance (*p* < 0.05). Combining traditional exercise with virtual reality (VR) strategies further enhances efficacy: initial traditional exercise promotes BDNF elevation and enhanced neuroplasticity, followed by VR-EI to strengthen brain network connectivity. Regular monitoring of brain network dynamics and emotional changes via EEG and behavioral assessments (e.g., PANAS scale) is recommended, with task content dynamically adjusted to accommodate individual neurofunctional variations.

### 4.5 Multiband brain mechanisms underlying mood improvement through virtual reality exercise

Correlation analyses further confirmed that the increase in positive affect (PA) within the VR-EI group showed significant positive correlations with enhanced θ-band DAN and SN activity (r=0.38-0.46) and enhanced α-band SMN-SN, SN, and DMN activity (r=0.40-0.42). This preliminary establishes a direct neural-behavioral pathway: “multisensory immersive exercise - low-frequency band attention-significance-default mode network remodeling-rapid positive emotion improvement.” This finding suggests that immersive virtual reality exercise not only enhances positive emotions through multisensory stimulation but may also achieve this effect by modulating brain activity in specific frequency bands. Specifically, the interaction between the attentional network in the theta band and the default mode network in the alpha band may play a crucial role in emotional regulation. These findings align strongly with multiple recent acute exercise EEG studies in adults with depression, where a single aerobic session significantly enhances α-band frontal-limbic coupling and elevates positive emotions within 20–30 minutes (42); immersive VR exercise further amplifies the attention-emotion network integration effect in the θ/α bands. This study is the first to validate this pathway in adolescents with subclinical depression. Notably, VR-EI demonstrated particularly pronounced regulatory advantages in low-frequency bands (theta/alpha),potentially due to the multimodal (visual-auditory-proprioceptive) synchronized stimulation provided by virtual natural landscapes activating broader dopamine-norepinephrine pathways (43). Adolescent brains, being in a peak period of cortical-limbic system plasticity (44), exhibit heightened sensitivity to multisensory stimulation. The study further indicates that VR-EI not only improves emotional states in the short term but may also exert positive effects on long-term mental health. By enhancing functional connectivity within brain networks, VR-EI may promote the development of emotional regulation capabilities, offering a novel intervention approach for adolescents. Notably, the immersion and pleasure experienced by participants during VR-EI may further amplify the exercise’s positive effects. This immersive experience effectively reduces psychological stress, enhances self-efficacy, and consequently fosters the development of psychological resilience.

This study preliminarily demonstrated the efficacy of virtual reality emotional intervention (VR-EI) in improving brain network function and emotional states among students with depressive symptoms through static brain network analysis and the Positive and Negative Affect Schedule (PANAS). However, the study has the following limitations: First, intervention protocol constraints. The intervention cycle was relatively short, with a fixed single-session duration of 15 minutes and uniformly set moderate exercise intensity (50%-80% HRmax). While this standardized protocol facilitated variable control, it failed to explore dose-response effects across different cycles, durations, or intensity gradients, nor did it fully account for the potential impact of individual fitness variations on intervention response. Second, implementation methods and generalizability: The study was conducted in a highly standardized laboratory setting under continuous researcher supervision. While this ensured intervention fidelity, the generalizability and sustainability of these effects in real-world settings—such as regular school curricula or home environments—remain to be validated. Finally, the specific influence of virtual reality technology: as previously noted, VR technology itself is a multi-faceted composite. The specific combination of VR device model, rendering engine, and scene content used in this study constitutes a particular “technology package.” Variations in other technical parameters—such as latency, resolution, or interactive feedback modes—may produce distinct effects on brain activity, thereby limiting the direct transferability of these findings to other VR technology configurations.

## 5 Conclusions

Depressive symptoms in adolescents are associated with multifrequency brain network dysregulation. Combining exercise intervention with virtual reality technology (VR-EI) optimizes key brain network connectivity and activity in the theta and alpha bands through multisensory stimulation. Its mood-enhancing effects surpass those of conventional exercise, offering a promising new strategy for personalized intervention in adolescent depression. Future research should focus on refining intervention parameters, exploring personalized protocol design, and validating long-term efficacy in more realistic settings.

## Data Availability

The raw data supporting the conclusions of this article will be made available by the authors, without undue reservation.
